# Can the Life-History Strategy Explain the Success of the Exotic Trees *Ailanthus altissima* and *Robinia pseudoacacia* in Iberian Floodplain Forests?

**DOI:** 10.1371/journal.pone.0100254

**Published:** 2014-06-17

**Authors:** Pilar Castro-Díez, Guillermo Valle, Noelia González-Muñoz, Álvaro Alonso

**Affiliations:** 1 Department of Life Sciences (U.D. Ecology), University of Alcalá, Alcalá de Henares, Madrid, Spain; 2 Laboratory of Systematic Ecology and Evolution, University of Paris-Sud, Paris, France; University of A Coruña, Spain

## Abstract

*Ailanthus altissima* and *Robina pseudoacacia* are two successful invasive species of floodplains in central Spain. We aim to explain their success as invaders in this habitat by exploring their phenological pattern, vegetative and sexual reproductive growth, and allometric relations, comparing them with those of the dominant native tree *Populus alba*. During a full annual cycle we follow the timing of vegetative growth, flowering, fruit set, leaf abscission and fruit dispersal. Growth was assessed by harvesting two-year old branches at the peaks of vegetative, flower and fruit production and expressing the mass of current-year leaves, stems, inflorescences and infrutescences per unit of previous-year stem mass. Secondary growth was assessed as the increment of trunk basal area per previous-year basal area. *A. altissima* and *R. pseudoacacia* showed reproductive traits (late flowering phenology, insect pollination, late and long fruit set period, larger seeds) different from *P. alba* and other native trees, which may help them to occupy an empty reproductive niche and benefit from a reduced competition for the resources required by reproductive growth. The larger seeds of the invaders may make them less dependent on gaps for seedling establishment. If so, these invaders may benefit from the reduced gap formation rate of flood-regulated rivers of the study region. The two invasive species showed higher gross production than the native, due to the higher size of pre-existing stems rather than to a faster relative growth rate. The latter was only higher in *A. altissima* for stems, and in *R. pseudoacacia* for reproductive organs. *A. altissima* and *R. pseudoacacia* showed the lowest and highest reproductive/vegetative mass ratio, respectively. Therefore, *A. altissima* may outcompete native *P. alba* trees thanks to a high potential to overtop coexisting plants whereas *R. pseudoacacia* may do so by means of a higher investment in sexual reproduction.

## Introduction

In the last decades, the scientific interest in explaining causes and consequences of biological invasions has drastically increased [1]–[4], partly due to the social and economic impact of some invasion events [5], [6]. In the case of plants, many studies have identified functional traits associated to the invasive success, such as high growth rate, low cost of tissue production, resprouting ability, small seed size, profuse seed production, long flowering period or N-fixation ability [7]–[10]. In addition, different properties of ecosystems promoting invasions have been reported, such as high and fluctuating resource availability, high frequency of disturbances, biogeographic isolation, existence of empty niches or high spatial/temporal heterogeneity [11]–[16]. However, most studies agree in the context-dependency of plant traits and of the ecosystem properties associated to invasions [10], [17], [18]. In particular, it has been suggested that the chances of success, as well as the magnitude of the impact, largely depends on the functional difference between the exotic species and the prevailing native vegetation [2], [19], [20].

Recent studies have emphasized the relevance of phenological differences between exotic and native plants as a mechanism favouring invasions [21]–[24]. For instance, a different phenology allows an exotic species to benefit from resources (radiation, pollinators, nutrients) that are temporally available [21], [25] and contributes to stabilize the interactions between the exotic and the native species [26]–[28]. In addition, a different phenology may also provide exotics certain competitive advantage, either because earlier phenology allows sequestering resources first [29], because a later phenology allows higher production after escaping from competition by early-senescent native plants [24], [25], [30]–[32], or because a more extended phenology provides greater access to resources [10], [33], [34] (see Wolkovich & Clealand, 2011 for a review). Other features recently recorded as relevant for the success of exotic plants as invaders are vegetative growth and sexual reproduction. A high vegetative growth rate has been frequently found in invasive species [8], [35]. This trait is largely responsible for the competitive capacity of a plant species [36]. High seed production has been also shown as a relevant contributor to invasiveness, as it allows an efficient spread [37], [38]. However, there is a trade-off between investment in vegetative growth and sexual reproduction [39], [40]. The balance between both plant functions is very dependent on the selective forces of the environment where the species have evolved. In this sense, classical r-K and CSR hypotheses predict that frequently-disturbed environments promote investment to sexual reproduction at the expense of growth, while stable environments, where competition is the main selective force, foster growth over sexual reproduction [36], [41], [42]. Therefore a sudden change in the disturbance regime may reduce the fitness of the native species and promote invasions by exotic plants with growth/reproduction balance more suited to the new conditions.

In the Mediterranean part of Europe, floodplains are one of the habitats more susceptible to exotic plant invasions [43]. This is partly because river floods bring about some of the properties of invasion-prone environments mentioned above, such as high and fluctuating resource availability, and spatial and temporal heterogeneity [44], [45]. Besides, rivers are efficient dispersal agents for exotic seeds [44], [46], [47]. Finally, human management of rivers and floodplains (e.g. flow regulation, removal of riparian forests, deepening of water tables) can alter selective forces in riparian forests at a rate that cannot be tracked by genetic changes in long-life plants, such as trees. Thus, these human actions may facilitate plant invasion by reducing native plant fitness under the new conditions and by providing conditions that directly benefit invading species [48], [49].

In Mediterranean riparian forests of central Spain, the native *Populus alba* L. dominates clay-rich basic soils of middle or low river stretches, where it coexists with other less abundant native trees, such as *Salix alba* L., *Populus nigra* L., *Fraxinus angustifolia* L. and *Ulmus minor* Mill. [50]. Since the 19^th^ century these species also co-occur with naturalized exotic trees, such as *Ailanthus altissima* (Mill.) Swingle and *Robinia pseudoacacia* L., which are native to E-China and the Appalachian Mountains (USA), respectively [51]–[53]. These exotic trees are efficient colonizers of disturbed sites, but they also colonize riparian forest and displace natural vegetation [51]–[54]. Both species possess many of the functional traits conferring invasive success, such as N-fixation ability in *R. pseudoacacia*, profuse seed production and fast growth in *A. altissima* and vegetative reproduction by root suckering in both species [52]–[54]. Besides, the two species are well known for their ecological impacts on the invaded ecosystems. For instance, *A. altissima* may increase soil fertility [55], [56] and suppress native vegetation by competition and/or allelopathic effects [52], [57], [58]. *R. pseudoacacia* may increase the input of atmospheric N into the soil [59], [60], but the high lignin content of its leaves make their decomposition slower than that of co-occurring native deciduous leaves of riparian forests [61]–[63]. This may explain the low colonization of *R. pseudoacacia* leaves by aquatic invertebrates [63]. Accordingly, both species are included in the list of the top 100 more aggressive invaders in Europe [64]. However, little effort has been done so far to understand the context-specific factors explaining the success of these invasive species in *P. alba*-dominated riparian forests of Central Spain.

We aim to explain the invasive success of *A. altissima* and *R. pseudoacacia* in the above described community by comparing their life-history strategies with that of the dominating native tree in the recipient community, *P. alba*. We hypothesize that (1) the exotic species differ in phenology from the dominating natives [21]; (2) a different organ phenology allows the invaders to achieve larger relative production of that organ, due to earlier resource sequestration and/or longer resource uptake period; and (3) given that flood regulation has decreased the disturbance frequency of floodplains, successful invaders are expected to be pre-adapted to more stable environments by a higher vegetative to reproductive mass ratio than the native species.

## Methods

### Study Species and Site

**Figure 1:**
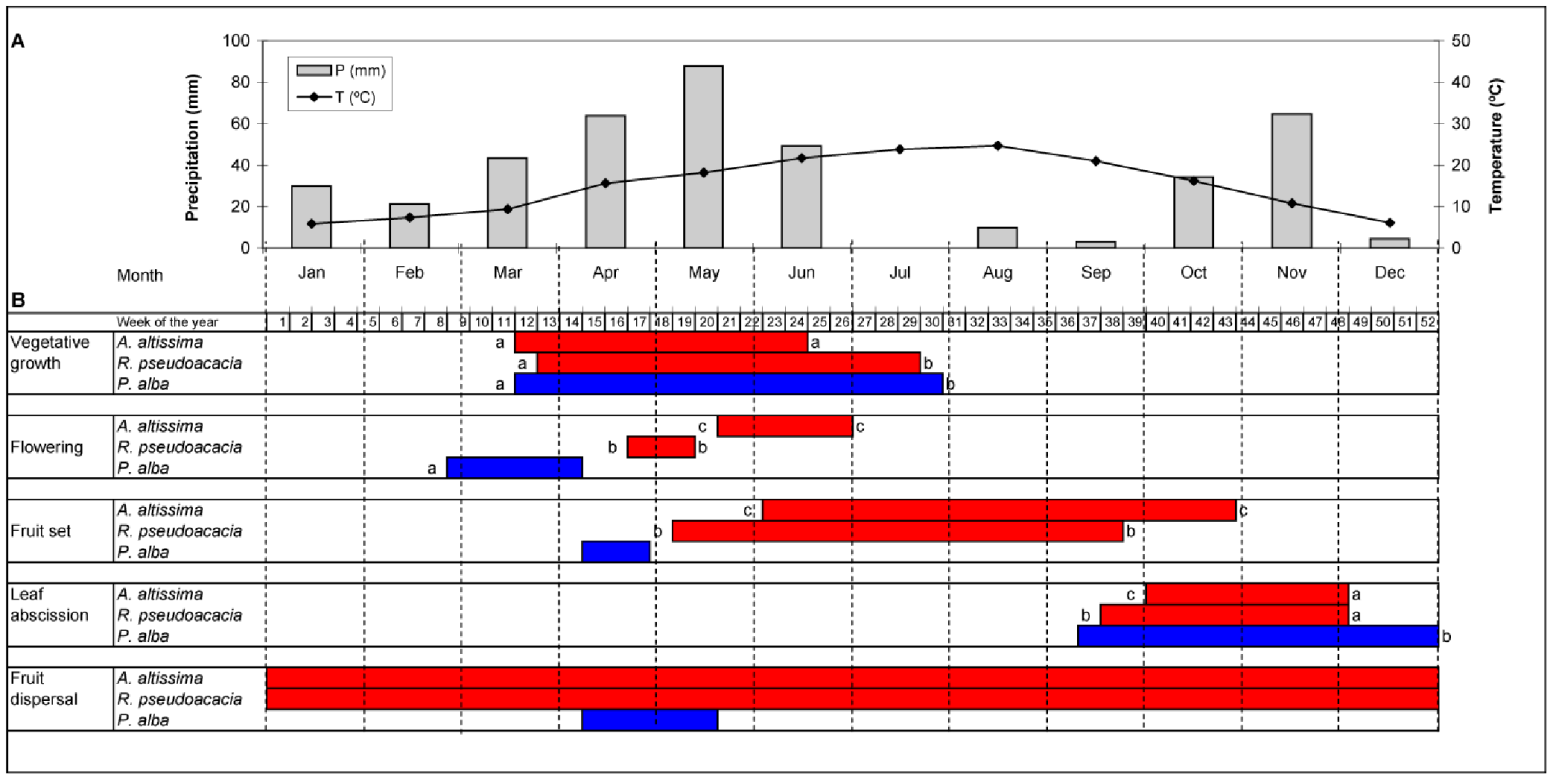
Climatic conditions and plant phenology. A) Climatic diagram showing monthly mean temperature and precipitation during 2011 (Torrejón de Ardoz weather station). B) Diagrams of phenological activity of the three species in 2011. Different letters across species for the beginning or end of each phenophase indicate significant differences after a pairwise Watson-Williams multisample test (*P*<0.05). Missing letters means that comparisons could not be performed for all species due the lack of variation between replicates.

This study was conducted in the floodplains of a medium-low stretch of the Henares River in central Spain (Province of Madrid), which is a public non protected area where no permit access is required. The study area spans over 22 km, from Alcalá de Henares to Mejorada del Campo. Environmental conditions and vegetation structure along this stretch are considered to be homogeneous [50]. Soils are luvisols and fluvisols [65] with a pH near 8 and a percentage of organic matter of 4.6–9 [61]. Altitude ranged from 554 to 602 m above sea level. Climate is continental Mediterranean with hot and dry summers and cold winters. Mean annual temperature and annual precipitation in the study area were 14.1°C and 528.5 mm in 2010 and 15.1°C and 411.1 mm in 2011 (Fig. 1) (data from “Torrejón de Ardoz” weather station, National Institute of Meteorology). In the study area the riparian forest is mostly constrained to a narrow band due to the occupation of floodplains by crops, industry and human settlements. The main dominant tree in the native community is *Populus alba*, which is accompanied by *S. alba* and to a minor extent, *P. nigra, F. angustifolia and U. minor* [50]. Among them, patches of the exotics *A. altissima* and *R. pseudoacacia* are frequent, mainly in human-disturbed areas [51]. *P. alba* has been found to be an exotic invasive species in other regions, such as Australia and South Africa [66], [67].

The three studied species, *Populus alba*, *A. altissima* and *R. pseudoacacia*, are considered as fast-growing pioneer trees which can reach up to 25 m of height [51], [52], [68], [69]. *P. alba* and *A. altissima* are dioecious, while *R. pseudoacacia* is monoecious but self-pollination is prevented by a hairy collar below the stigma [68]. The native *P. alba* is wind-pollinated [70] while the two exotics are pollinated by insects [51], [52]. Seeds are primarily dispersed by wind in the three species, although only *P. alba* and *A. altissima* have specific structures assisting wind-dispersal [52], [71]. While seed viability last only 2–4 weeks in *P. alba*, the two exotics are able to form seed banks, both in the soil and in the plant canopy [52], [72].

### Phenological Monitoring

In January 2011, before any sign of budbreak, we selected 10–11 healthy adult trees per species to performed phenological observations. After flowering, we selected 5 and 8 additional trees in *A. altissima* and *P. alba*, respectively, to have representation of both sexes. Mean ± SE trunk diameter at breast height (DBH) of selected trees were 34.7±6.2, 28.0±3.3 and 33.5±4.0 cm for *A. altissima, R. pseudoacacia* and *P. alba*, respectively, which was representative of the average tree size in the studied populations. Monitoring of phenology spanned from 7^th^ February 2011 until 12^th^ April 2012, when fruit dispersal of *A. altissima* was completed. Every 1–3 weeks, depending on the activity of the plant, we noted whether the following phenophases were active or not: leaf development, flowering, fruit set, fruit dispersal, leaf abscission. Phenophases were considered active in a tree when they were easily observed at naked eye in at least 5% of the crown. These data are available at Table S1.

### Branch Production

In January 2010 we selected 6 adult trees of *R. pseudoacacia*, 6 female and 6 male adult trees of *A. altissima*. This year we could not sample *P. alba* due to technical reasons. To minimize the risk of branch collection interfering with tree growth, in January 2011 we selected 6 new trees per species and sex plus 6 female adult trees of *P. alba* (the sex of the selected trees could not be identified before flowering, which occurred in the second sampling date, see Table 1). In every selected tree we measured DBH and performed 2–3 harvests of 5–10 two-year-old branches, coinciding with the time of maximum leaf, flower and fruit production (see Table 1). Branches were firstly dissected, separating current year (*t*) and previous-year (*t*-1) cohorts, and then each cohort was separated into different organs, oven-dried (>48 h at 60°C) and weighted (stem mass-*S*, leaf mass-*L*, inflorescence mass-*FL* and infrutescence mass-*FR*). To be representative of the full tree, current-year FL and FR values were multiplied by the proportion of branches in the canopy holding flowers or fruits. This proportion was estimated at the time of flower and fruit sampling as follows: we divided the canopy of each tree in four quarters and randomly selected 10 branches in each quarter. The proportion of the selected branches holding flowers/fruits was considered to be representative for the full tree. To correct for the allometric effects of previous-year shoot mass on current-year production, the oven-dried weight of every current-year fraction (*S_t_, L_t_, FL_t_* and *FR_t_*) was divided by that of the previous-year stem (*S_t−1_*) bearing the fraction. In this way we obtained the relative production of stems (*S_t_/S_t−1_*), leaves (*L_t_/S_t−1_*), inflorescences (*FL_t_/S_t−1_*) and infrutescences (*FR_t_/S_t−1_*). In the case of *P. alba*, the six initially selected trees failed to produce fruits, so they were replaced by 6 additional trees from the same population for the last sampling of fruit production. These data are available at Table S2.

**Table 1:** Details of the branch production sampling.

							Dates of collection (day/month)		
Species	Origin	Sampling year	Sex	No. Sampled trees	No. sampled branches/tree	DBH (cm)	Leaf production	Flower production	Fruit production
*A. altissima*	I	2010	male	6	5	49.76±7.33	1–8 June	1–8 June	–
		2010	female	6	5	22.16±7.10	16–23 June	16–23 June	18–20 October
		2011	male	6	8	12.40±1.16	31 May	31 May	–
		2011	female	6	8	29.50±6.46	31 May–2 June	31 May-2 June	15–20 September
*R. pseudoacacia*	I	2010		5	5	29.63±5.53	23 May	23 May	19 October
		2011		6	10	37.83±4.93	27 April	27 April	22 August
*P. alba*	N	2011	female	6	10	38.65±10.29	29 April	25 March	–
		2011	female	6	10	24.80±5.88	–	–	29 April

Branch production sampling details for the studied species *Ailanthus altissima*, *Robinia pseudoacacia* and *Populus alba*: origin of the species (I-invasive, N-native); year of sampling, tree sex (in the case of dioecious species), number of sampled trees and branches per tree, mean±SE of trunk diameter at breast height (DBH), and dates of branch collection for each peak of organ production.

### Secondary Growth

In February 2012, before the onset of the new growing season, 3–4 radial wood samples were extracted at 1.30 m of trunk height using an increment core borer (Haglöf, Sweden) from the same trees used in 2011 for branch production. Wood samples were glued on wooden holders and sanded until tree-rings were clearly visible under a binocular microscope. The width of the last two xylem rings, corresponding to 2010 (*RW_10_*) and 2011 (*RW_11_*), was measured with an accuracy of 1/100 mm using a LINTAB measuring table and the software TSAP [73]. At the same height where cores were extracted we measured trunk perimeter (*P*) to derive the trunk radius (*R = P/2π*) and assessed the average cork thickness (*C_th_*) with a cork calliper (Haglöf, Sweden) in four equidistant points around the trunk. Using these data we estimated the trunk basal area in 2011 excluding the cork (*BA_2011_ = π(R-C_th_*)^2^), the 2010 basal area (*BA_2010_ = π(R-C_th_-RW_11_*)^2^ and the 2009 basal area (*BA_2009_ = π(R-C_th_-RW_11_-RW_10_*)^2^). The basal area increment of each tree in 2010 and 2011 *(ΔBA_t_*) was the difference between current- and previous-year basal areas. These data are available at Table S3.

### Statistical Analysis

Phenological data were analysed using circular statistics with Oriana 2.0 package. Dates of beginning and end of each phenophase (expressed as weeks since January the 1^st^ and transformed into angles of a 52-week circle) were pair-wise compared across species using the multisample Watson-Williams F test [74].

In previous analyses we found that some growth variables differed between years in some species. For instance, in *R. pseudoacacia* a liner mixed model, using year as factor, DBH as covariate and tree as random factor, revealed that *S_t_/S_t−1_* was significantly larger in 2010 than in 2011 (0.37 and 0.19 gg^−1^ respectively, *t* = 4.45, *p* = 0.002). Therefore, we decided to perform all analyses separately for 2010 and 2011. Although in 2010 we lack of native control, we decided to compare the two invasive species to check the extent to which the patterns found for them were consistent across years.

The allometric relations of *S_t−1_* with *S_t_*, *L_t_*, *FL_t_* and *FR_t_* were fitted using standard major axis estimation (SMA) after log-transforming both variables [75]. SMA lines were tested for different slopes between sexes in *A. altissima* and across species using the likelihood ratio statistic (LRS). When cross-species differences were detected, we performed pairwise comparisons, lowering the significance level to 0.017, following a Bonferroni correction for multiple comparisons [76]. When slopes did not differ across species, they were additionally tested for equal elevation and for shifts along the common axis, using the Wald test [75]. Analyses were performed using the “smart” package in R 3.0.2.

*S_t_, L_t_, FL_t_, FR_t_,S_t_/S_t−1_, L_t_/S_t−1_, FL_t_/S_t−1_* and *FR_t_/S_t−1_* were compared among species, using linear mixed models, with species as fix factor, DBH as covariate and tree as random factor. When species was significant, pairwise comparisons were performed using a Tukey post-hoc test. In the case of *A. altissima*, variables were also compared among sexes (fix factor), using DBH as additional fix factor and tree as random factor. When necessary, variables were log-, 0.25- or 0.01-power-transformed to achieve homoscedasticity and normal distribution of residuals. Analyses were performed using R 3.0.2 and packages “nmle” and “multcomp”.

To assess the relative investment in vegetative growth vs. sexual reproduction, we represented in a plane defined by the mean vegetative (leaves+stems) and reproductive (inflorescences+infrutescences) mass produced per previous-year stem, each species, sex (in the case of *A. altissima*) and each year. Species above the 1∶1 line had a relative higher investment in sexual reproduction while species below this line invested more in vegetative growth.

The allometric relation between basal area increment *(ΔBA_t_*) and previous-year basal area *(BA_t−1_*) was fitted using standard major axis estimation (SMA). SMA lines were tested for different slope, elevation and shift across species as explained above.

## Results

### Phenological Differences Across Species

Vegetative growth occurred mainly in spring in the three species, largely coinciding with the months of higher precipitation (Fig. 1). Although the three species started at a similar date, *A. altissima* arrested growth significantly earlier than *R. pseudoacacia* and *P. alba* (Fig. 1). Flowering phenology largely contrasted across species, being the earliest in *P. alba* (March), intermediate in *R. pseudoacacia* (late April–mid May), and the latest in *A. altissima* (June) (Fig. 1). Accordingly, fruit set was earlier and shorter in *P. alba* (April), followed by *R. pseudoacacia* (May–September) and by *A. altissima* (June–October) (Fig. 1). Leaf abscission occurred mostly in autumn, coinciding with the temperature drop, although *A. altissima* started later (early October), *P. alba* ended later (late December), and *R. pseudoacacia* was intermediate (Fig. 1). Finally, fruit dispersal occurred all over the year in the invaders, while in *P. alba* it was concentrated between April and May (Fig. 1).

### Branch Production

In *A. altissima* the slope of the allometric lines did not differ between sexes (LRS<0.5 and P>0.36 in all cases), but the *L_t_* − *S_t−1_* line showed higher elevation in females, whereas the *FL_t_- S_t−1_* line showed higher elevation in males (Fig. S1). Similarly, *FL_t_/S_t−1_* tended to be marginally higher in males (linear mix model effect F-value for sex = 3.38, *P* = 0.10, Table S4). This means that, for a given unit of previous year stem, females produce more leaf mass but less flower mass than males. The rest of variables were not affected by sex (Table S4).

**Figure 2:**
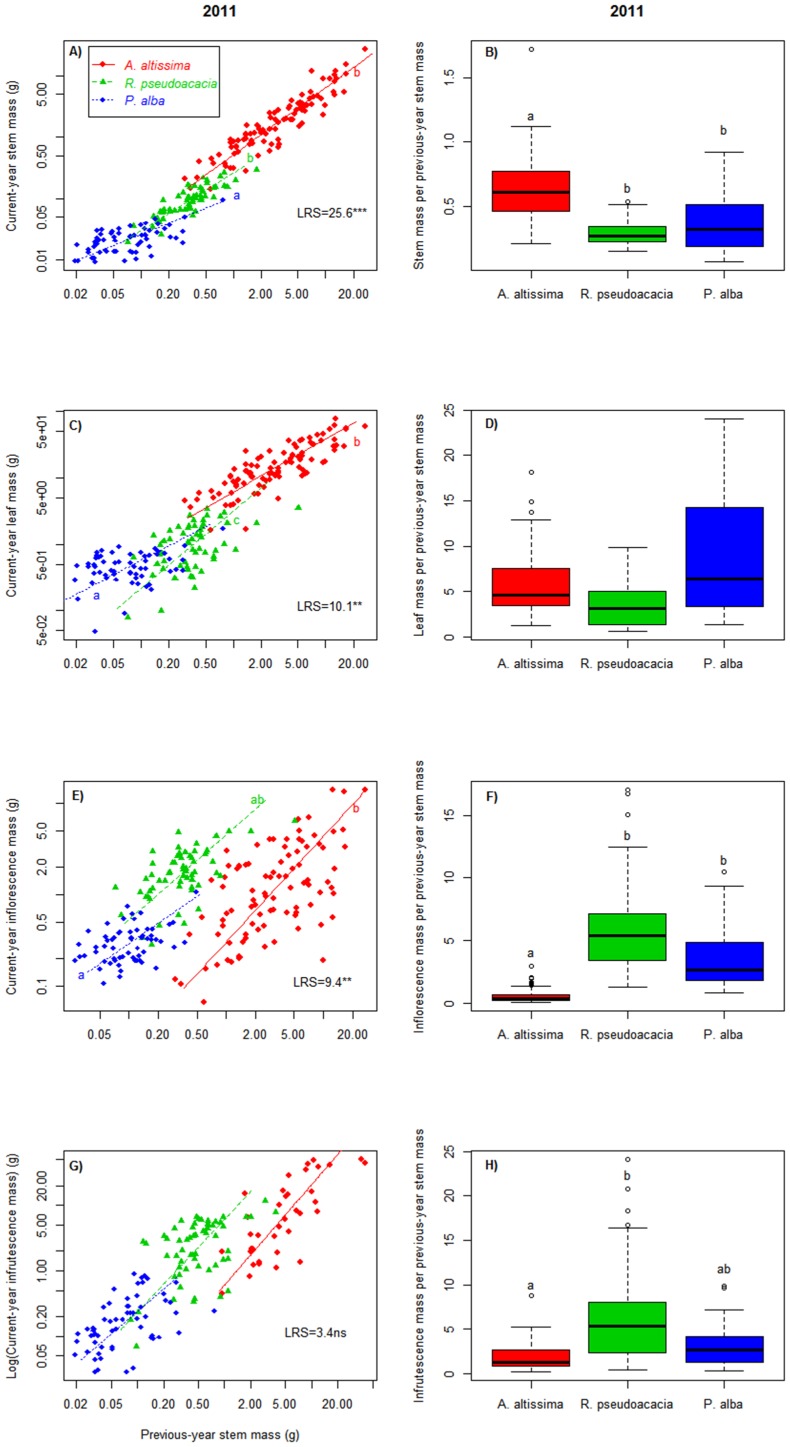
Species branch production in 2011. Left: Allometric relationships between previous-year stem mass and current year stem (A), leaf (C), inflorescence (E) and infrutescence (G) mass (note the log-scale of both axes). The Likelihood Ratio Statistic (LRS) comparing slopes across species and its significance is shown in each chart. Different letters indicate significant slope differences (*P*<0.017, after Bonferroni correction). Right: relative production of current-year stem (B), leaf (D), inflorescence (F) and infrutescence (H) mass, expressed per unit of previous-year stem mass. Different letters across species indicate significant differences (Tukey test, see Table 3 for more details).

The mass of every branch organ in either cohort was the largest in *A. altissima* and the smallest in *P. alba*, although *FL_t_* and *FR_t_* did not differ between *A. altissima* and *R. pseudoacacia* in 2011 and 2010, respectively (Table 2). However, when organ mass was relativized by *S_t−1_*, *A. altissima* tended to show larger relative production only for stems, but lower for reproductive organs (Fig. 2 right, Fig. S2). In 2011, linear mixed models showed that *S_t_/S_t−1_* was the largest in *A. altissima* and similar between *P. alba* and *R. pseudoacacia* (Fig. 2B). *L_t_/S_t−1_* was similar across species, being marginally smaller in *R. pseudoacacia* than in *P. alba* (*P* = 0.07). By contrast, *FL_t_/S_t−1_* and *FR_t_/S_t−1_* followed the ranking *R. pseudoacacia* ≥ *P. alba* ≥ *A. altissima* (Fig. 2F, H). This ranking was consistent independently of whether any or both sexes were considered in *A. altissima* (data not shown). In 2010 comparisons between *A. altissima* and *R. pseudoacacia* showed similar trends, but less often significant (Fig. S2). Among all analyses, DBH was only significant in the case of *S_t_/S_t−1_* of 2011, where it showed a negative slope (i.e. thicker trees tended to produce less stem mass). In 2011, the random factor (tree) explained between 37 and 57% of the variance not explained by the fixed factors, while in 2010 this proportion varied from 6% to 83% (Table 3).

**Table 2:** Gross branch production per species, year and organ type.

Sp	Year	*S_t_* (g)	*L_t_*(g)	*FL_t_*	*FR_t_*
*A. altissima*	2010	3.23±0.63^A^	22.79±3.53^A^	6.49±0.76^A^	5.71±1.88^A^
*R. pseudoacacia*		0.15±0.02^B^	1.81±0.19^B^	1.11±0.14^B^	1.39±0.24^A^
*A. altissima*	2011	2.94±0.40^a^	18.59±1.51^a^	1.98±0.27^a^	13.68±2.52^a^
*R. pseudoacacia*		0.11±0.01^b^	1.19±0.11^b^	2.01±0.16^a^	3.20±0.31^b^
*P. alba*		0.02±0.00^c^	0.51±0.03^c^	0.32±0.02^b^	0.24±0.03^c^

Mean±SE gross mass of stems (*S_t_*), leaves (*L_t_*), inflorescences (*FL_t_*) and infrutescences (*FR_t_*) produced per previous year stem in the invasive (*Ailanthus altissima* and *Robinia pseudoacacia*) and native (*Populus alba*) trees, in 2010 and in 2011. Different letters in the same variable and year indicate significant differences between species after a linear mixed model, with species and DBH as fix factors and individual as random factor, followed by a post-hoc Tukey test.

**Table 3:** Comparison of relative production across species.

		Species		DBH			
Variable	Year	*F*-value	*P*-value	*F*-value	*P*-value	Tree	Residual
*S_t_/S_t−1_*	2010	11.35	0.007	1.36	0.270	0.06	0.94
*H_t_/S_t−1_*		0.02	0.891	3.37	0.096	0.40	0.60
*FL_t_/S_t−1_*		1.42	0.264	0.94	0.358	0.83	0.17
*FR_t_/S_t−1_*		2.79	0.133	0.61	0.459	0.68	0.32
*S_t_/S_t−1_*	2011	123.45	0.000	49.81	0.039	0.41	0.59
*H_t_/S_t−1_*		36.66	0.046	0.90	0.356	0.57	0.43
*FL_t_/S_t−1_*		557.19	0.000	0.02	0.900	0.37	0.63
*FR_t_/S_t−1_*		50.46	0.026	31.09	0.103	0.46	0.54

Results of the linear mixed model assessing the effects of species and tree diameter at breast height (DBH) on current-year stem (*S_t_*), leaf (*L_t_*), inflorescence (*FL_t_*) and infrutescence (*FR_t_*) biomass per unit of previous-year stem mass (*S_t−1_*). Tree was included as random factor. The last two columns show the proportion of variance not explained by the fixed factors that was explained by the random factor (Tree) or not (Residual). All variables were 0.25-power-transformed, except *S_t_/S_t−1_* which was log-transformed.

The allometric relationships revealed that species differed in the gain of current year organ mass per unit of *S_t−1_* increase. In the case of *S_t_* and *FL_t_*, this gain was the largest in *A. altissima* and the smallest in *P. alba* (see slopes in Fig. 2A and 2E). In the case of *L_t_*, the gain was the lowest in *P. alba*, which showed the smallest slope, *R. pseudoacacia* showing larger slope than *A. altissima* (Fig. 2C). In the case of infrutescences, slopes of SMA lines were similar across species, although *A. altissima* exhibited smaller elevation than the other species (Wald statistic = 52.3 P<0.001), i.e. this species produced less infrutescence mass for a given value of previous-year stem mass (Fig. 2G).

**Figure 3:**
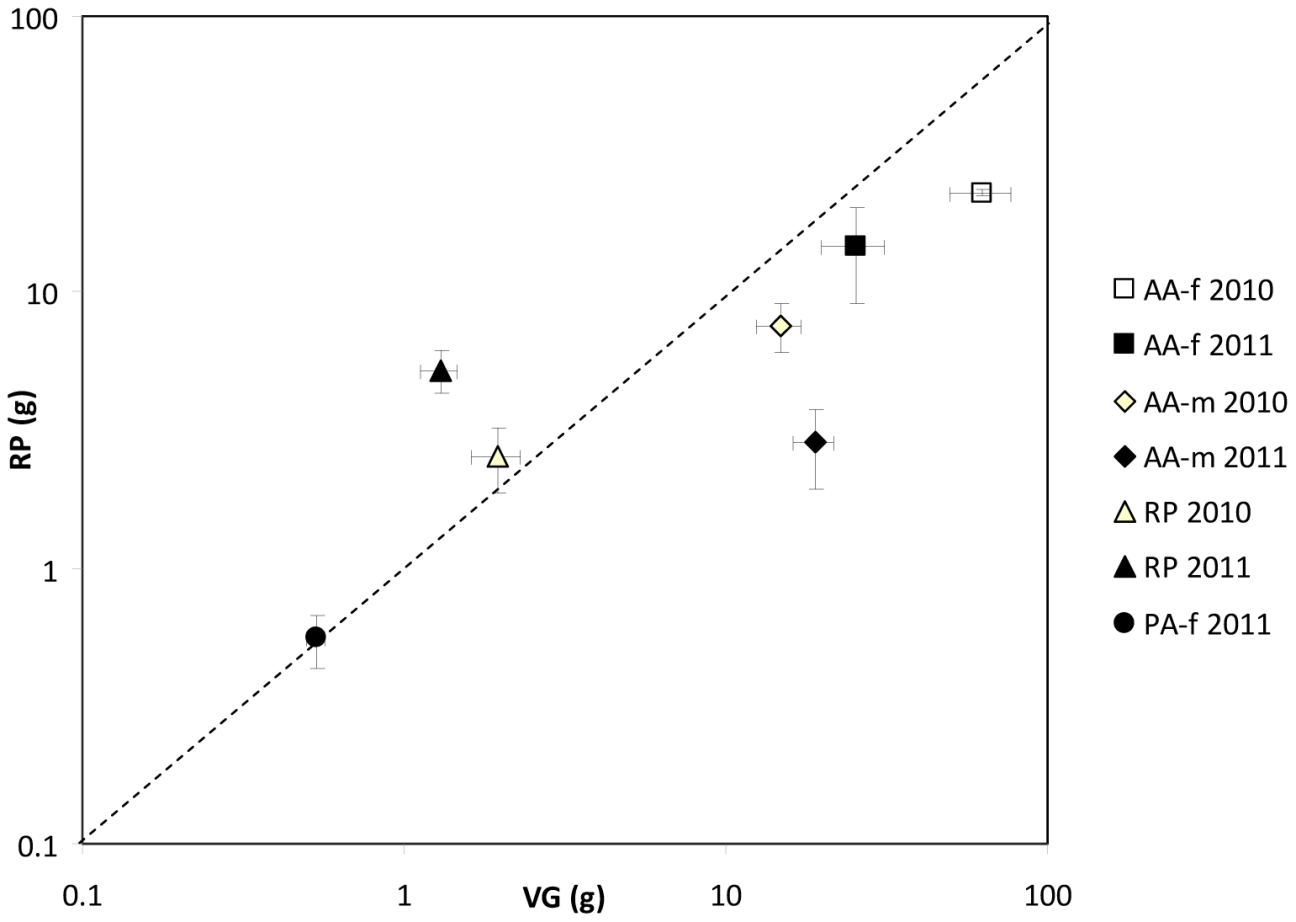
Relationship between vegetative and reproductive production. Relationship between mean (±SE) vegetative mass (VG = leaf+stem mass) and the mean (±SE) reproductive mass (RP = inflorescence+infrutescence mass) produced per previous-year stem in each species (AA- *Ailanthus altissima*, RP- *Robinia pseudoacacia*, PA- *Populus alba*), each sex (f-female, m-male) and each study year (2010 and 2011). The dashed line indicates the 1∶1 relation between VG and RP. Species lying above this line invested more in reproduction, while species below the line invested more in vegetative growth. (Note the log-scale of both axes).

The relationship between vegetative and reproductive mass per previous-year stem was c.a. 1∶1 in female *P. alba* trees of 2011. *R. pseudoacacia* invested more in sexual reproduction, either in 2010 and 2011, while *A. altissima* invested more in vegetative growth, disregard the sex and the year (Fig. 3).

### Secondary Growth

**Figure 4:**
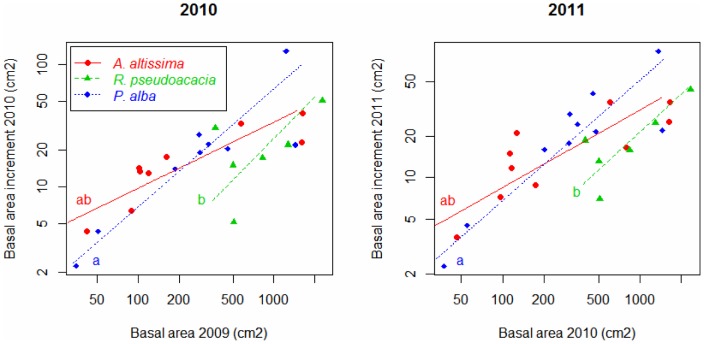
Tree basal area increment. Allometric relationship between current-year basal area increment and previous-year basal area for 2010 (left) and 2011 (right). Each dot corresponds to a different tree. Different letters indicate significant difference of elevation across species (pairwise Wald test for multiple comparison, *P*<0.017, after Bonferroni correction). Slopes did not significantly differ across species (see Table 4).

The annual increment of trunk basal area allometrically increased with previous-year basal area in all species (Fig. 4). The slope of SMA lines tended to be smaller for *A. altissima* than for the remaining species, but this difference was marginally significant in 2010 and non-significant in 2011 (Table 4). The elevation of the SMA line was significantly smaller for *R. pseudoacacia* than for *P. alba* (Fig. 3, Table 4), which means that, at equal previous-year basal area, *R. pseudoacacia* increased less its basal area than *P. alba*. *Ailanthus altissima* did not differ in the elevation of its SMA line from the remaining species. Results were consistent between years (Fig. 4, Table 4).

**Table 4:** Comparison of the allometric relation between trunk growth and area across species.

	2010		2011	
Comparison of:	Statistic	*P*-value	Statistic	*P*-value
Slopes (likelihood ratio test)	4.838	0.089	2.782	0.249
Elevation (Wald test)	5.184	0.075	10.520	0.005
Shift along common axes (Wald test)	2.452	0.293	2.090	0.352

Cross-species comparison of the allometric relation between tree basal area increment and previous-year basal area, adjusted with standard major axes (SMA). The shown tests compared slope, elevation and shift along a common axe of SMA lines between species (SMA lines are shown in Fig. 4).

## Discussion

### Is there a Phenological Offset between the Invaders and the Native?

We expected *A. altissima* and *R. pseudoacacia* to differ from *P. alba* in phenology (hypothesis 1), as a mechanism to exploit temporal empty niches, to reduce competition and to promote coexistence [22], [23], [25], [30], [77]. This hypothesis was only supported for reproductive growth, which occurred later and during a longer period in the invaders than in the native tree (see Fig. 1). Given that flowering and fruiting phenology –at monthly resolution– where available in the literature for other native woody species, we also compared the invaders phenology with that of the most frequent native woody plants of the studied floodplains. Most native trees (i.e. *Populus nigra, Salix alba* and *Ulmus minor*) show a reproductive phenology similar to *P. alba* (early flower and fruit set), only *F. angustifolia* showing a pattern in between *P. alba* and the invaders (early flower but autumn fruit ripening [70]). By contrast, the reproductive phenology of the tree invaders resembles more to that of several native entomophilous riparian shrubs / vines, such as *Cornus sanguinea* L., *Humulus lupulus* L., *Ligustrum vulgare* L. or *Rubus ulmifolius* Schott, which flower from April/May to July/August, and extend fruit set until autumn [70].

The period when a plant flowers is determined by the interaction between genetic factors and environmental variables (e.g. water availability, temperature or day length) [78]–[81], or pollinator phenology [82], [83]. An evidence of the genetic control of flowering time is the fact that invasive plants tend to keep their native flowering phenology after being transferred to a climatically different region [77]. One genetic factor that constrains flowering phenology is the architecture of buds [84]. Species which bloom before leafing-out, such as *P. alba,* necessarily possess separated reproductive (R) and vegetative (V) buds, which allows any combination of vegetative and reproductive phenology [84], [85]. By contrast, *A. altissima* and *R. pseudoacacia* enclose vegetative and reproductive primordia within the same buds (VR buds), which forces these species to start shoot elongation and leaf expansion before bloom [84] (see Fig. S3). Among environmental factors, summer drought is a strong selective force for phenology in Mediterranean regions, where flowering mostly occurs in spring [84], [86]–[88]. However, the presence of a water table in the floodplains probably makes this factor less limiting, since late-spring/summer flowering is not uncommon among native undercanopy shrubs and vines of the studied region [70]. If the climatic filter is not so strong, pollination type may be a more relevant driver for flowering phenology. Wind-pollinated plants, such as *P. alba,* and the other native trees (*P. nigra, S. alba, U. minor* and *F. angustifolia*) [70], have been found to bloom earlier than animal-pollinated plants [83], as wind pollination is more efficient when plants are still bare of leaves. By contrast, *A. altissima* and *R. pseudoacacia*, like the above mentioned native shrubs/vines, are insect-pollinated and present late flowering [52]–[54], [70]. In this case, a successful pollination requires blooming to occur at a time with high insect activity, which coincides with the months of warmer temperature [89], [90].

### What are the Benefits of an Offset Phenology for the Invaders?

The niche for insect-pollinated late-flowering trees seems to be empty in the studied native community and successfully colonized by *A. altissima* and *R. pseudoacacia*. The invaders may benefit from the reproductive asynchrony with native trees due to a reduced competition for the resources demanded for flowering and fruit set, i.e. nutrients, water and pollinators, which in *A. altissima* and *R. pseudoacacia* are mainly honeybees, beetles and other nectar- and pollen-feeding generalist insects [52], [68], [91]. In fact, a shortage of pollinators has been found to limit fruit production in many invasive plants, including *R. pseudoacacia* [92]–[94]. Given that all native trees in the recipient community are wind-pollinated [70], *A. altissima* and *R. pseudoacacia* only have to face competition for pollinators by the sub-dominant undercanopy entomophilous shrubs and vines. Moreover, the latter species may facilitate exotic tree invasion by providing an established pollinator community, as found before [93], [95]. *A. altissima* and *R. pseudoacacia* also contrasted with *P. alba* in the year-round seed dispersal period. Seed viability is very short in *P. alba* and the remaining native trees of the studied community [70], [72], [96] but it extends for one year or more in the invaders, which allows them to form seed banks, both in the canopy and in the soil [97]–[100]. In this way these species widen the temporal window for sexual reproduction and reduce seed loss in years with poor conditions for germination [101], [102]. Another remarkable difference between the invaders and the native is the longer fruit setting period of the former. Our hypothesis 2 predicted that a more extended organ phenology would allow a higher relative production of that organ. However, against this prediction, we found that the relative fruit production of *P. alba* was not significantly different from any of the invaders (Fig. 2H). Alternatively, previous studies found a relation between fruit set duration and seed mass [103]. This was true for our species, as seed mass of the long fruit-set invaders (*A. altissima* = 14–25 mg, *R. pseudoacacia* = 16–20 mg [104]) were almost two orders of magnitude larger than the short fruit-set native *P. alba* (0.1–0.6 mg [70], [72], [104]). Large seeds have been found to produce larger seedlings, to have more chances to grow through the thick debris layer of deciduous forests, and to expand leaves earlier –before canopy closure-, therefore having more chances to survive in the undercanopy [105]. Although *A. altissima* and *R. pseudoacacia* possess many traits of pioneer trees and gap-colonizers [52], [68], [71], [104], their larger seeds may make them less dependent on gaps for sexual reproduction than the tiny-seeded *P. alba*. Moreover, gaps in riparian forest are becoming less frequent due to flow regulation, which decreases the frequency of gap-opening extraordinary floods [48], [49] and expands the potential regeneration niche for large-seeded plants. Most of the remaining native trees of the native community are also tiny-seeded (e.g. *P. nigra*, *S. alba* and *U. minor*) and only *F. angustifolia* possesses seeds larger than those of the invaders [70], [72], [104]. By contrast, other exotic trees, such as *Acer negundo* L. and *Elaeagnus angustifolia* L, which are potentially invasive in Spanish riparian forests [51], also possess large seeds [104]. Therefore native tree richness of the studied riparian forest might decline in the future at the expense of exotic tree richness if the dynamic of regulated rivers is not restored.

### Vegetative and Reproductive Growth of Invasive and Native Trees

Branch gross production was the highest and the lowest in *A. altissima* and *P. alba*, respectively, and intermediate in *R. pseudoacacia*, either for stems, leaves, inflorescences or infrutescences (Table 4). This result suggests a clear competitive advantage of the invaders (especially *A. altissima*) over the native at the adult stage. However, when plant production was relativized per unit of previous-year stem mass (i.e., relative production), differences between *R. pseudoacacia* and *P. alba* tended to disappear (vegetative and reproductive relative productions were marginally lower and higher in *R. pseudoacacia*, respectively) and *A. altissima* only retained a superior relative production for stems but showed the lowest reproductive relative production (Fig. 2B, F, H). Moreover, secondary growth relativized per unit of pre-existing basal area was the lowest in *R. pseudoacacia* and similar between *A. altissima* and *P. alba* (Fig. 4). This suggests that the observed superior gross production of the invaders was due to a larger pre-existing biomass, rather than to a faster growth, and the larger pre-existing biomass may be due to a faster growth at early ontogenetic stages. In line with this argument, annual tree height increment of *A. altissima* in different parts of the invaded range has been reported to be as large as 2 m in 1–2 year-old seedlings growing under favourable conditions [106], [107] and to decline to 8 cm or less in 20–25 year-old trees [52]. Similarly, in its native area *R. pseudoacacia*, has been reported to show a fast growth rate in the early life stages which sharply decreases at an age of only 10–20 years [71].

The larger relative production of stems (*L_t_/S_t−1_*) found in *A. altissima* agrees with the above reports of great potential for height growth [106], [107]. Moreover, the trend of *A. altissima* to have a less steep allometric relation between basal area increment and trunk area than the other species (see slopes in Fig. 4), suggests that *A. altissima* prioritize primary over secondary growth. Such a strategy may help this species to quickly overtop other coexisting plants and thus to escape from the canopy shade that it cannot tolerate [52], [108]. One functional property behind the high growth potential of *A. altissima* may relate to its ability to optimize light capture by combining physiological attributes of high- and low-irradiance adapted plants [109]. The low vegetative relative production of *R. pseudoacacia* was observed for both leaf and stem mass production (Fig. 2B, 2D), as well as for secondary growth (Fig. 4). This unexpected result for a species which is considered as a successful invader in Spain [51] may be attributed to different non-exclusive reason. First, in the study region *R. pseudoacacia* usually shows open crowns, sparse leaves and many dead branches, suggesting that trees are affected by any disease [110]. Second, given the positive association between secondary growth and April-June precipitation [111], the drastic summer decrease of rainfall typical of Mediterranean regions (see Fig. 1) may hamper the realization of its potential growth. Third, *R. pseudoacacia* growth rate may drastically decline with tree age according to the short-life and pioneer strategy described for this species [68], [71], so that the relative low growth capacity observed may be an ontogenetic effect. By contrast, this species exhibited the largest relative reproductive production, which may contribute to a great potential to spread.

Our third hypothesis predicted lower ratio of reproductive to vegetative annual mass production in the invaders than in the native as a pre-adaption to the decreased rate of gap formation resulting from flood regulation. In *P. alba*, this ratio was close to 1∶1 for female trees, although we can expect it to be slightly lower for males, as found in other dioecious species [39]. According to our hypothesis, *A. altissima* showed the lowest ratio, indicating that vegetative growth was prioritized over sexual reproduction, and in line with the great growth potential reported for this species [106]–[108]. However, contrary to our hypothesis, *R. pseudoacacia* showed the highest reproductive to vegetative mass ratio, which suggests a great potential to colonize new sites, but not so great to compete with other plants. The prioritization to sexual reproduction found in *R. pseudoacacia* is consistent with reports indicating that vegetative growth notably declines after 10–20 years [71], while fecundity might not decline before 30–40 years [94].

Altogether, our growth results suggest that both *A. altissima* and *R. pseudoacacia* may outcompete the native *P. alba* due to their large mass acquired at early stages. Later on, *A. altissima* would retain a great height growth capacity, which would allow it to overtop other coexisting plants. However, *R. pseudoacacia* would suffer a rapid decline in vegetative growth rate, but retain a high potential for fruit production which would make this species more efficient to colonize new sites than to compete with coexisting adult trees of *P. alba*.

## Conclusions

*Ailanthus altissima* and *R. pseudoacacia* show reproductive traits (late flowering phenology, insect-pollination, long fruit set period, relatively large seeds) different to those of the tree species dominating the studied riparian forest community of central Spain. These differences may help them to occupy an empty reproductive niche, and then benefit from a reduced competition for the resources required by flower/fruit development and for pollinators. Moreover, the long fruit set period of invaders and the associated larger seed mass may make them less dependent on gaps for seedling establishment than the tiny-seeded native tree *P. alba*. Such ability may help the invaders to expand their reproductive niche in flood-regulated rivers of the study region. The two invasive species showed a superior gross production than the native which was due to a higher size of pre-existing stems rather than to a faster relative growth rate. At the studied adult stage *A. altissima* and *R. pseudoacacia* showed the lowest and highest reproductive/vegetative mass ratio, respectively. Therefore, *A. altissima* might outcompete the native trees thanks to a higher vegetative growth whereas *R. pseudoacacia* may do so by means of a higher investment in sexual reproduction. Altogether, our findings suggest that preserving the natural flood regime of the river is the best strategy to favour the native trees over the two invaders in this case study. Given the great vegetative production of the invaders at adult stage, especially in the case of *A. altissima*, an efficient management should focus on early detection and prevention of establishment. Further information on the growth potential of all native and invasive tree species at different ontogenetic stages would help to predict the dynamics of this invaded riparian forest community.

## Supporting Information

Figure S1 Allometric relationships between previous-year stem mass and current year leaf (left) and inflorescence (right) mass in *A. altissima* (data of 2011). Different symbols and lines represent different sexes. The Wald statistic indicates a significant difference in line elevation. No significant difference was found between slopes.
(TIF)

Figure S2 Left- Allometric relationships between previous-year stem mass and current year stem (A), leaf (C), inflorescence (E) and infrutescence (G) mass (note the log-scale of both axes). The Likelihood Ratio Statistic (LRS) comparing slopes across species and its significance is shown in each chart. When slopes were equal, we also show results for the Wald satistics (WS) comparing elevation and shift along a common slope. Right- current-year stem (B), leaf (D), inflorescence (F) and infrutescence (H) mass per unit of previous-year stem mass. Different letters across species indicate significant differences (Linear mixed model, species and DBH being the fix factors and tree the random factor). Data from 2010 collection.
(TIF)

Figure S3 Left- Picture of a current-year shoot of *Ailanthus altissima*, showing that the same current-year stem bears leaves and inflorescences, which were derived from the same VR bud (see text) (April 2009). Centre- Picture of two recently opened buds of *Robinia pseudoacia*. It can be observed that both leaves and inflorescence derived from the same VR buds (March 2009). Right- Picture of a female inflorescence of *Populus alba*. It can be seen that the inflorescence grew on the previous-year stem from a R bud, while the apical vegetative (V) bud, enclosing the current-year vegetative organs, is still close (March 2009). Pictures by the authors.
(TIF)

Table S1 Phenological data of the studied species.
(XLSX)

Table S2 Branch production data of the studied species.
(XLSX)

Table S3 Secondary growth of the studied species.
(XLSX)

Table S4 Comparison of shoot production between sexes in *Ailanthus altissima*.
(DOC)

## References

[1] van Kleunen M, Dawson W, Schlaepfer D, Jeschke JM, Fischer M (2010) Are invaders different? A conceptual framework of comparative approaches for assessing determinants of invasiveness. Ecology Letters 13: 947–958. 20576028 10.1111/j.1461-0248.2010.01503.x

[2] Castro-Díez P, Godoy O, Alonso A, Gallardo A, Saldaña A (2014) What explains variation in the impacts of exotic plant invasions on the nitrogen cycle? A meta-analysis. Ecology Letters 17: 1–12. 24134461 10.1111/ele.12197

[3] Pyšek P, Jarosik V, Hulme PE, Pergl J, Hejda M, et al (2012) A global assessment of invasive plant impacts on resident species, communities and ecosystems: the interaction of impact measures, invading species’ traits and environment. Global Change Biology 18: 1725–1737.

[4] Vilà M, Espinar JL, Hejda M, Hulme PE, Jarosik V, et al (2011) Ecological impacts of invasive alien plants: a meta-analysis of their effects on species, communities and ecosystems. Ecology Letters 14: 702–708. 21592274 10.1111/j.1461-0248.2011.01628.x

[5] Pimentel D, Zuniga R, Morrison D (2005) Update on the environmental and economic costs associated with alien-invasive species in the United States. Ecological Economics 52: 273–288.

[6] Andreu J, Vila M, Hulme PE (2009) An Assessment of Stakeholder Perceptions and Management of Noxious Alien Plants in Spain. Environmental Management 43: 1244–1255. 19214625 10.1007/s00267-009-9280-1

[7] Pyšek P, Richardson DM (2007) Traits associated with invasiveness in alien plants: where do we stand? In: Nentwig W, editor. Biological Invasions. Berlin Heidelberg: Springer-Verlag. 97–125.

[8] van Kleunen M, Weber E, Fischer M (2010) A meta-analysis of trait differences between invasive and non-invasive plant species. Ecology Letters 13: 235–245. 20002494 10.1111/j.1461-0248.2009.01418.x

[9] Daehler CC (2003) Performances’s comparisons of co-occurring native and alien invasive plants: implications for conservation and restoration. Annual Review of Ecology and Systematics 34: 183–211.

[10] Lloret F, Médail F, Brundu G, Camarda I, Moragues E, et al (2005) Species attributes and invasion success by alien plants on Mediterranean islands. Journal of Ecology 93: 512–520.

[11] Davis MA, Grime JP, Thompson K (2000) Fluctuating resources in plant communities: a general theory of invasibility. Journal of Ecology 88: 528–534.

[12] Burke MJW, Grime JP (1996) An experimental study of plant community invasibility. Ecology 77: 776–790.

[13] Mack RN (2003) Phylogenetic constraint, absent life forms, and preadapted alien plants: a prescription for biological invasions. International Journal of Plant Sciences 164: S183–S196.

[14] Crawley MJ, Harvey PH, Purvis A (1996) Comparative ecology of the native and alien floras of the British Isles. Philosophical Transactions of the Royal Society B-Biological Sciences 351: 1251–1259.

[15] Melbourne BA, Cornell HV, Davies KF, Dugaw CJ, Elmendorf S, et al (2007) Invasion in a heterogeneous world: resistance, coexistence or hostile takeover? Ecology Letters 10: 77–94. 17204119 10.1111/j.1461-0248.2006.00987.x

[16] Catford JA, Jansson R, Nilsson C (2009) Reducing redundancy in invasion ecology by integrating hypotheses into a single theoretical framework. Diversity and Distributions 15: 22–40.

[17] Alpert P, Bone E, Holzapfel C (2000) Invasiveness, invasibility and the role of environmental stress in the spread of non-native plants. Perspectives in Plant Ecology Evolution and Systematics 3: 52–66.

[18] Dawson W, Burslem D, Hulme PE (2009) Factors explaining alien plant invasion success in a tropical ecosystem differ at each stage of invasion. Journal of Ecology 97: 657–665.

[19] D’Antonio CM, Corbin JD (2003) Effects of plant invaders on nutrient cycling: Using models to explore the link between invasion and development of species effects. In: Canham CD, Cole JJ, Lauenroth WK, editors. Models in Ecosystem Science. Princeton (NJ): Princeton University Press. 363–384.

[20] Fargione J, Brown CS, Tilman D (2003) Community assembly and invasion: An experimental test of neutral versus niche processes. Proceedings of the National Academy of Sciences of the United States of America 100: 8916–8920. 12843401 10.1073/pnas.1033107100 PMC166413

[21] Wolkovich EM, Cleland EE (2011) The phenology of plant invasions: a community ecology perspective. Frontiers in Ecology and the Environment 9: 287–294.

[22] Hooper DU, Dukes JS (2010) Functional composition controls invasion success in a California serpentine grassland. Journal of Ecology 98: 764–777.

[23] Willis CG, Ruhfel BR, Primack RB, Miller-Rushing AJ, Losos JB, et al (2010) Favorable climate change response explains non-native species’ success in Thoreau’s Woods. Plos One 5. 10.1371/journal.pone.0008878 PMC2811191 20126652

[24] Godoy O, Levine JM (2014) Phenology effects on invasion success: insights from coupling field 2 experiments to coexistence theory. Journal of Ecology: (in press). 10.1890/13-1157.1 24804456

[25] Dietz H, Ullmann I (1997) Phenological shifts of the alien colonizer *Bunias orientalis*: Image-based analysis of temporal niche separation. Journal of Vegetation Science 8: 839–846.

[26] Chesson P (2000) Mechanisms of maintenance of species diversity. Annual Review of Ecology and Systematics 31: 343–+.

[27] Adler PB, HilleRisLambers J, Levine JM (2007) A niche for neutrality. Ecology Letters 10: 95–104. 17257097 10.1111/j.1461-0248.2006.00996.x

[28] MacDougall AS, Gilbert B, Levine JM (2009) Plant invasions and the niche. Journal of Ecology 97: 609–615.

[29] Seabloom EW, Harpole WS, Reichman OJ, Tilman D (2003) Invasion, competitive dominance, and resource use by exotic and native California grassland species. Proceedings of the National Academy of Sciences of the United States of America 100: 13384–13389. 14595028 10.1073/pnas.1835728100 PMC263823

[30] Fridley JD (2012) Extended leaf phenology and the autumn niche in deciduous forest invasions. Nature 485: 359–U105. 22535249 10.1038/nature11056

[31] Arianoutsou M, Bazos I, Delipetrou P, Kokkoris Y (2010) The alien flora of Greece: taxonomy, life traits and habitat preferences. Biological Invasions 12: 3525–3549.

[32] Crawley MJ (1987) What makes a comunity invasible? In: Gray AJ, Crawley MJ, Edwards PJ, editors. Colonization, succession and stability: Blackwell Sci. Pub. 429–453.

[33] Gerlach JD, Rice KJ (2003) Testing life history correlates of invasiveness using congeneric plant species. Ecological Applications 13: 167–179.

[34] Cadotte MW, Lovett-Doust J (2001) Ecological and taxonomic differences between native and introduced plants of southwestern Ontario. Ecoscience 8: 230–238.

[35] Grotkopp E, Rejmánek M (2007) High seedling relative growth rate and specific leaf area are traits of invasive species: phylogenetically independent contrasts of woody angiosperms. American Journal of Botany 94: 526–532. 21636422 10.3732/ajb.94.4.526

[36] Grime JP (1977) Evidence for the existence of three primary strategies in plants and its relevance to ecological and evolutionary theory. The American Naturalist 111: 1169–1194.

[37] Richardson DM (2006) *Pinus*: a model group for unlocking the secrets of alien plant invasions? Preslia 78: 375–388.

[38] Colautti RI, Grigorovich IA, MacIsaac HJ (2006) Propagule pressure: A null model for biological invasions. Biological Invasions 8: 1023–1037.

[39] Milla R, Castro-Díez P, Maestro-Martínez M, Montserrat-Martí G (2006) Costs of reproduction as related to the timing of phenological phases in the dioecious shrub *Pistacia lentiscus* L. Plant Biology. 8: 103–111. 16435274 10.1055/s-2005-872890

[40] Obeso JR (2002) The costs of reproduction in plants. The New Phytologist 155: 321–348. 10.1046/j.1469-8137.2002.00477.x 33873312

[41] MacArthur RH, Wilson EO (1967) The theory of island biogeography. Princeton, New Jersey: Princeton Univ. Press. 203 p.

[42] Pianka ER (1970) On *r*- and *k*- selection. The American Naturalist 104: 592–597.

[43] Chytrý M, Maskell LC, Pino J, Pyšek P, Vilà M, et al (2008) Habitat invasions by alien plants: a quantitative comparison between Mediterranean, subcontinental and oceanic regions of Europe. Journal of Applied Ecology 45: 448–458.

[44] Tickner DP, Angold PG, Gurnell AM, Mountford JO (2001) Riparian plant invasions: hydrogeomorphological control and ecological impacts. Progress in Physical Geography 25: 22–52.

[45] Hood WG, Naiman RJ (2000) Vulnerability of riparian zones to invasion by exotic vascular plants. Plant Ecology 148: 105–114.

[46] Tabacchi E, Planty-Tabacchi AM, Roques L, Nadal E (2005) Seed inputs in riparian zones: Implications for plant invasion. River Research and Applications 21: 299–313.

[47] Säumel I, Kowarik I (2013) Propagule morphology and river characteristics shape secondary water dispersal in tree species. Plant Ecology 214: 1257–1272.

[48] Catford JA, Downes BJ, Gippel CJ, Vesk PA (2011) Flow regulation reduces native plant cover and facilitates exotic invasion in riparian wetlands. Journal of Applied Ecology 48: 432–442.

[49] Glenn EP, Nagler PL (2005) Comparative ecophysiology of *Tamarix ramosissima* and native trees in western U.S. riparian zones. Journal of Arid Environments 61: 419–446.

[50] Lara F, Garillete R, Ramírez P (1996) Estudio de la vegetación de los ríos carpetanos de la cuenca del Jarama. Madrid: Centro de Estudios y Experimentación de Obras Públicas. Ministerio de Fomento. 270 p.

[51] Sanz Elorza M, Dana Sánchez ED, Sobrino Vesperinas E (2004) Atlas de las plantas alóctonas invasoras en España. Madrid: Dirección General para la Biodiversidad. Ministerio de Medio Ambiente. 384 p.

[52] Kowarik I, Saumel I (2007) Biological flora of Central Europe: *Ailanthus altissima* (Mill.) Swingle. Perspectives in Plant Ecology Evolution and Systematics 8: 207–237.

[53] Basnou C (2009) *Robinia pseudoacacia* L., black locust (Fabaceae, Magnolipphyta). In: DAISIE, editor. Handbook of alien species in Europe: Springer Publishers. 357.

[54] Basnou C, Vilà M (2009) *Ailanthus altissima* (Mill.) Swingle, tree of heaven (Simabouraceae, Magnoliphyta). In: DAISIE, editor. Handbook of alien species in Europe: Springer Publishers. 342.

[55] Gomez-Aparicio L, Canham CD (2008) Neighborhood models of the effects of invasive tree species on ecosystem processes. Ecological Monographs 78: 69–86.

[56] Vilà M, Tessier M, Suehs CM, Brundu G, Carta L, et al (2006) Local and regional assessment of the impacts of plant invaders on vegetation structure and soil properties of Mediterranean islands. Journal of Biogeography 33: 853–861.

[57] Heisey RM, Heisey TK (2003) Herbicidal effects under field conditions of *Ailanthus altissima* bark extract, which contains ailanthone. Plant and Soil 256: 85–99.

[58] Gómez-Aparicio L, Canham CD (2008) Neighbourhood analyses of the allelopathic effects of the invasive tree *Ailanthus altissima* in temperate forests. Journal of Ecology 96: 446–458.

[59] Tateno R, Tokuchi N, Yamanaka N, Du S, Otsuki K, et al (2007) Comparison of litterfall production and leaf litter decomposition between an exotic black locust plantation and an indigenous oak forest near Yan’an on the Loess Plateau, China. Forest Ecology and Management 241: 84–90.

[60] Rice SK, Westerman B, Federici R (2004) Impacts of the exotic, nitrogen-fixing black locust (*Robinia pseudoacacia*) on nitrogen-cycling in a pine-oak ecosystem. Plant Ecology 174: 97–107.

[61] Castro-Díez P, González-Muñoz N, Alonso A, Gallardo A, Poorter L (2009) Effects of exotic invasive trees on nitrogen cycling: a case study in Central Spain. Biological Invasions 11: 1973–1986.

[62] Castro-Díez P, Fierro-Brunnenmeister N, González-Muñoz N, Gallardo A (2012) Effects of exotic and native tree leaf litter on soil properties of two contrasting sites in the Iberian Peninsula. Plant and Soil 350: 179–191.

[63] Alonso A, González-Muñoz N, Castro-Díez P (2010) Comparison of leaf decomposition and macroinvertebrate colonization between exotic and native trees in a freshwater ecosystem. Ecological Research 25: 647–653.

[64] DAISIE (2009) Handbook of alien species in Europe; Drake JA, editor: Springer Publishers. 399 p.

[65] Monturiol F, Alcalá L (1990) Mapa de Asociaciones de suelos de la Comunidad de Madrid. Escala 1:200.000: CSIC y Comunidad de Madrid.

[66] Nel JL, Richardson DM, Rouget M, Mgidi TN, Mdzeke N, et al (2004) A proposed classification of invasive alien plant species in South Africa: towards prioritizing species and areas for management action. South African Journal of Science 100: 53–64.

[67] Herbarium WA (1998–2014) FloraBase—the Western Australian Flora. Department of Parks and Wildlife. http://florabase.dpaw.wa.gov.au/.

[68] Cierjacks A, Kowarik I, Joshi J, Hempel S, Ristow M, et al (2013) Biological Flora of the British Isles: *Robinia pseudoacacia* . Journal of Ecology 101: 1623–1640.

[69] Castroviejo S, Acedo C, Cirujano S, Laínz M, López González G, et al., editors (1993) Flora Iberica. Vol. 3. Madrid: Real Jardín Botánico, C. S. I. C. 730 p.

[70] Prada MA, Arizpe D, editors (2008) Riparian tree and shrub propagation handbook. An aid to riverine restoration in the Mediterranean region. Valencia: Generalitat Valenciana. 203 p.

[71] Boring LR, Swank WT (1984) The Role of Black Locust (*Robinia pseudoacacia*) in Forest Succession. Journal of Ecology 72: 749–766.

[72] Piotto B, Di Noi A, editors (2003) Seed propagation of Mediterranean trees and shrubs. Rome, Italy: APAT- Agency for the protection of the environment and for technical services. 120 p.

[73] Rinn F (1996) TSAP (Time series Analysis and Presentation) Version 3.0. Heidelberg, Germany.

[74] Zar JH (1984) Biostatistical analysis; edition S, editor: Prentice-Hall International, Inc.

[75] Warton DI, Wright IJ, Falster DS, Westoby M (2006) Bivariate line-fitting methods for allometry. Biological Reviews 81: 259–291. 16573844 10.1017/S1464793106007007

[76] Dunn OJ (1961) Multiple comparisons among means. Journal of the American Statistical Association 56: 52–&.

[77] Godoy O, Castro-Díez P, Valladares F, Costa-Tenorio M (2009) Different flowering phenology of alien invasive species in Spain: evidence for the use of an empty temporal niche? Plant Biology 11: 803–811. 19796357 10.1111/j.1438-8677.2008.00185.x

[78] Rathcke B, Lacey EP (1985) Phenological patterns of terrestrial plants. Annual Review of Ecology and Systematics 16: 179–214.

[79] Putterill J, Laurie R, Macknight R (2004) It’s time to flower: the genetic control of flowering time. Bioessays 26: 363–373. 15057934 10.1002/bies.20021

[80] Fox GA (1990) Components of flowering time-variation in a desert annual. Evolution 44: 1404–1423. 28564318 10.1111/j.1558-5646.1990.tb03835.x

[81] Hollister RD, Webber PJ, Bay C (2005) Plant response to temperature in Northern Alaska: Implications for predicting vegetation change. Ecology 86: 1562–1570.

[82] Wright SJ, Calderon O (1995) Phylogenetic patterns among tropical flowering. Journal of Ecology 83: 937–948.

[83] Bolmgren K, Eriksson O, Linder HP (2003) Contrasting flowering phenology and species richness in abiotically and biotically pollinated angiosperms. Evolution 57: 2001–2011. 14575322 10.1111/j.0014-3820.2003.tb00380.x

[84] Castro-Díez P, Montserrat-Martí G (1998) Phenological pattern of fifteen Mediterranean phanerohytes from *Quercus ilex* communities of NE-Spain. Plant Ecology 139: 103–112.

[85] Kozlowski TT (1971) Growth and development of trees; Kozlowski TT, editor. New York: Academic Press. 443 p.

[86] Orshan G, editor (1989) Plant pheno-morphological studies in Mediterranean type ecosystems. Dordrecht: Kluwer Acad. Pub. 404 p.

[87] Perez Latorre AV, Gavira O, Cabezudo B (2007) Ecomorphology and phenomorphology of mediterranean heathlands (SW Iberian peninsula). Phytocoenologia 37: 239–268.

[88] Godoy O, Richardson DM, Valladares F, Castro-Díez P (2009) Flowering phenology of invasive alien plant species compared with native species in three Mediterranean-type ecosystems. Annals of Botany 103: 485–494. 19033284 10.1093/aob/mcn232 PMC2707327

[89] Kudo G, Ida TY (2013) Early onset of spring increases the phenological mismatch between plants and pollinators. Ecology 94: 2311–2320. 24358716 10.1890/12-2003.1

[90] Iler AM, Inouye DW, Hoye TT, Miller-Rushing AJ, Burkle LA, et al (2013) Maintenance of temporal synchrony between syrphid flies and floral resources despite differential phenological responses to climate. Global Change Biology 19: 2348–2359. 23640772 10.1111/gcb.12246

[91] Jung SC, Matsushita N, Wu BY, Kondo N, Shiraishi A, et al (2009) Reproduction of a *Robinia pseudoacacia* population in a coastal *Pinus thunbergii* windbreak along the Kujukurihama Coast, Japan. Journal of Forest Research 14: 101–110.

[92] Parker IM (1997) Pollinator limitation of *Cytisus scoparius* (Scotch broom), an invasive exotic shrub. Ecology 78: 1457–1470.

[93] Sargent RD, Ackerly DD (2008) Plant-pollinator interactions and the assembly of plant communities. Trends in Ecology & Evolution 23: 123–130. 18262307 10.1016/j.tree.2007.11.003

[94] Masaka K, Yamada K, Koyama Y, Sato H, Kon H, et al (2010) Changes in size of soil seed bank in *Robinia pseudoacacia* L. (Leguminosae), an exotic tall tree species in Japan: Impacts of stand growth and apicultural utilization. Forest Ecology and Management 260: 780–786.

[95] Brown BJ, Mitchell RJ, Graham SA (2002) Competition for pollination between an invasive species (Purple loosetrife) and a native congener. Ecology 83: 2328–2336.

[96] Catalán Bachiller G (1991) Semillas de árboles y arbustos forestales. Madrid: Misnisterio de Agricultura Pesca y Alimentación. 392 p.

[97] Constan-Nava S, Bonet A (2012) Genetic variability modulates the effect of habitat type and environmental conditions on early invasion success of *Ailanthus altissima* in Mediterranean ecosystems. Biological Invasions 14: 2379–2392.

[98] Toole EH, Brown E (1946) Final results of the Duvel buried seed experiment. Jour Agric Res 72: 201–210.

[99] Masaka K, Yamada K (2009) Variation in germination character of *Robinia pseudoacacia* L. (Leguminosae) seeds at individual tree level. Journal of Forest Research 14: 167–177.

[100] Kota NL, Landenberger RE, McGraw JB (2007) Germination and early growth of Ailanthus and tulip poplar in three levels of forest disturbance. Biological Invasions 9: 197–211.

[101] Thompson K (1992) The functional ecology of seed banks. In: Fenner M, editor. The ecology of regeneration of plant communities. Wallingford, U.K.: CAB Int. 231–258.

[102] Ooi MKJ (2012) Seed bank persistence and climate change. Seed Science Research 22: S53–S60.

[103] Castro-Díez P, Montserrat-Martí G, Cornelissen JHC (2003) Trade-offs between phenology, relative growth rate, life form and seed mass among 22 Mediterranean woody species. Plant Ecology 166: 117–129.

[104] González-Muñoz N, Castro-Díez P, Godoy O (2014) Lack of superiority of invasive over co-occurring native riparian tree seedling species. Biological Invasions 16: 269–281.

[105] Seiwa K, Kikuzawa K (1996) Importance of seed size for the establishment of seedlings of five deciduous broad-leaved tree species. Vegetatio 123: 51–64.

[106] Hu SY (1979) *Ailanthus* . Arnoldia 39: 29–50.

[107] Pan E, Bassuk N (1986) Establishment and distribution of *Ailanthus altissima* in the urban environment. Journal of Environmental Horticulture 41: 1–4.

[108] Knapp LB, Canham CD (2000) Invasion of an old-growth forest in New York by *Ailanthus altissima*: sapling growth and recruitment in canopy gaps. Journal of the Torrey Botanical Society 127: 307–315.

[109] Hamerlynck EP (2001) Chlorophyll fluorescence and photosynthetic gas exchange responses to irradiance of Tree of Heaven (*Ailanthus altissima*) in contrasting urban environments. Photosynthetica 39: 79–86.

[110] González-Muñoz N, Castro-Díez P, Parker IM (2013) Differences in nitrogen use between native and exotic tree species: predicting impacts on invaded ecosystems. Plant and Soil 363: 319–329.

[111] Koretsune S, Fukuda K, Chang ZY, Shi FC, Ishida A (2009) Effective rainfall seasons for interannual variation in delta C-13 and tree-ring width in early and late wood of Chinese pine and black locust on the Loess Plateau, China. Journal of Forest Research 14: 88–94.

