# Supplementary figures and images for: Can the Life-History Strategy Explain the Success of the Exotic Trees Ailanthus altissima and Robinia pseudoacacia in Iberian Floodplain Forests?
Source: PLoS One. 2014 Jun 17;9(6):e100254. doi: 10.1371/journal.pone.0100254 (PMC4061096; doi:10.1371/journal.pone.0100254)

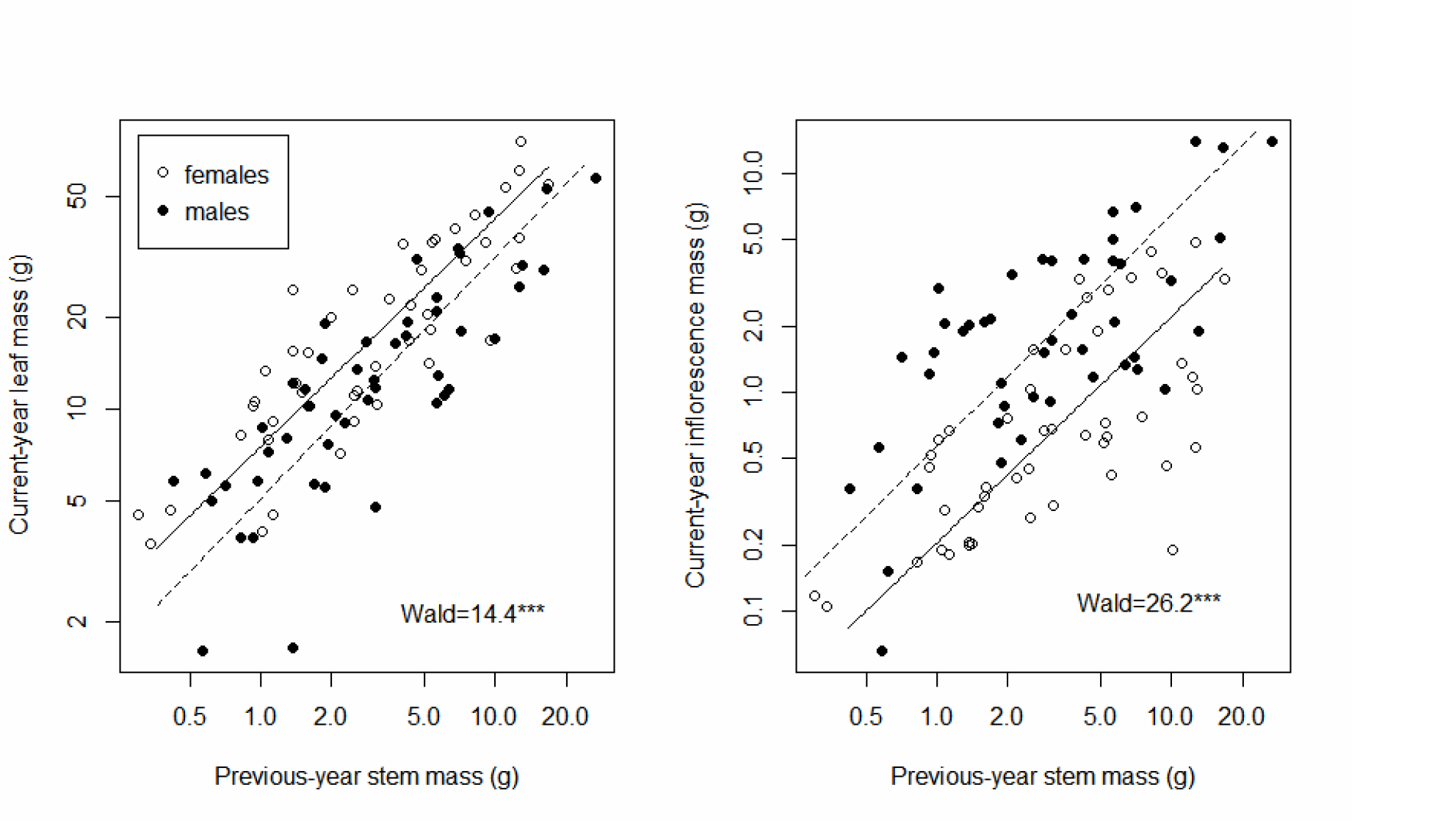

Supplement: Figure S1 — Allometric relationships between previous-year stem mass and current year leaf (left) and inflorescence (right) mass in A. altissima (data of 2011). Different symbols and lines represent different sexes. The Wald statistic indicates a significant difference in line elevation. No significant difference was found between slopes. (TIF) [file pone.0100254.s001.tif]

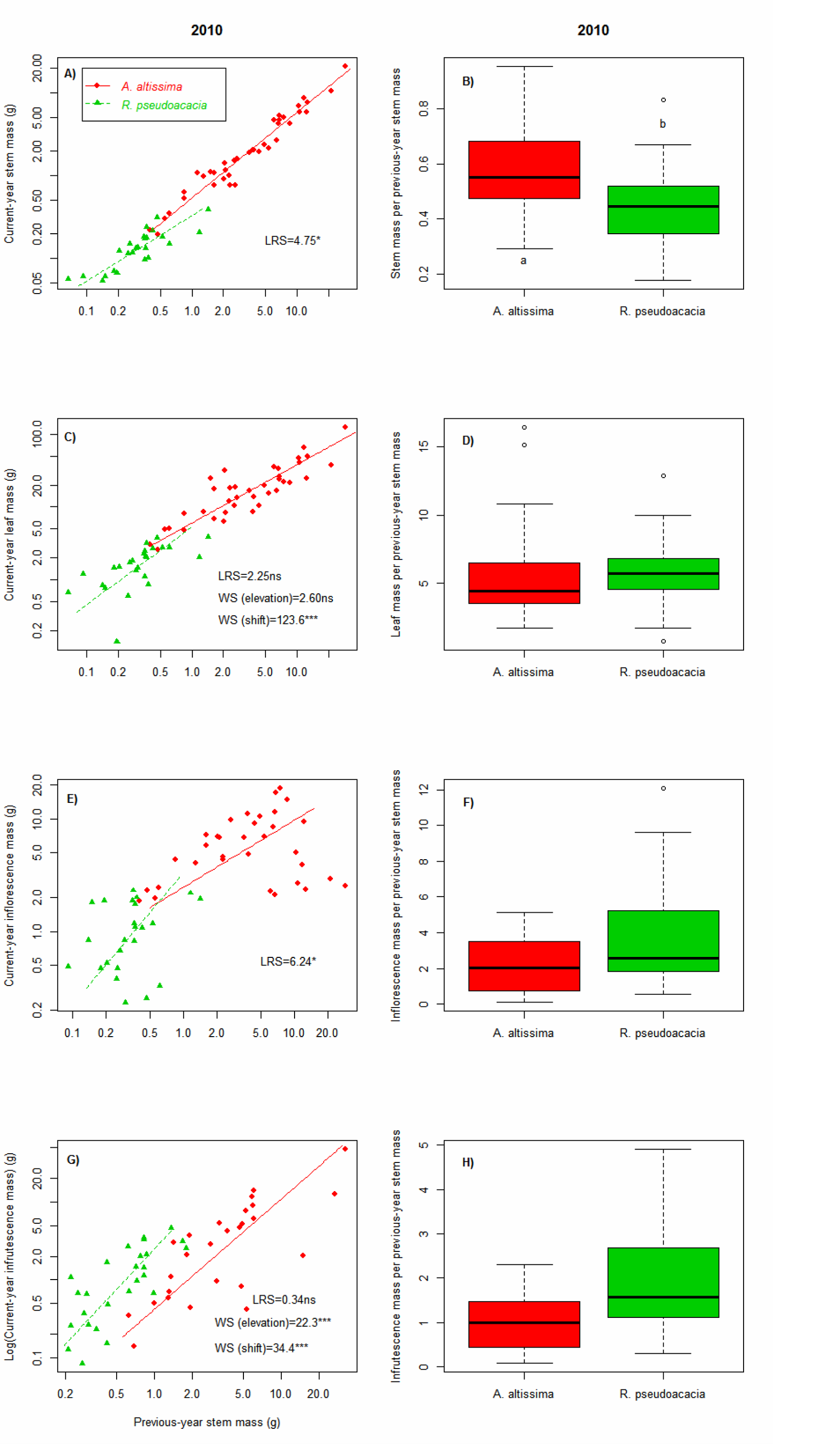

Supplement: Figure S2 — Left- Allometric relationships between previous-year stem mass and current year stem (A), leaf (C), inflorescence (E) and infrutescence (G) mass (note the log-scale of both axes). The Likelihood Ratio Statistic (LRS) comparing slopes across species and its significance is shown in each chart. When slopes were equal, we also show results for the Wald satistics (WS) comparing elevation and shift along a common slope. Right- current-year stem (B), leaf (D), inflorescence (F) and infrutescence (H) mass per unit of previous-year stem mass. Different letters across species indicate significant differences (Linear mixed model, species and DBH being the fix factors and tree the random factor). Data from 2010 collection. (TIF) [file pone.0100254.s002.tif]

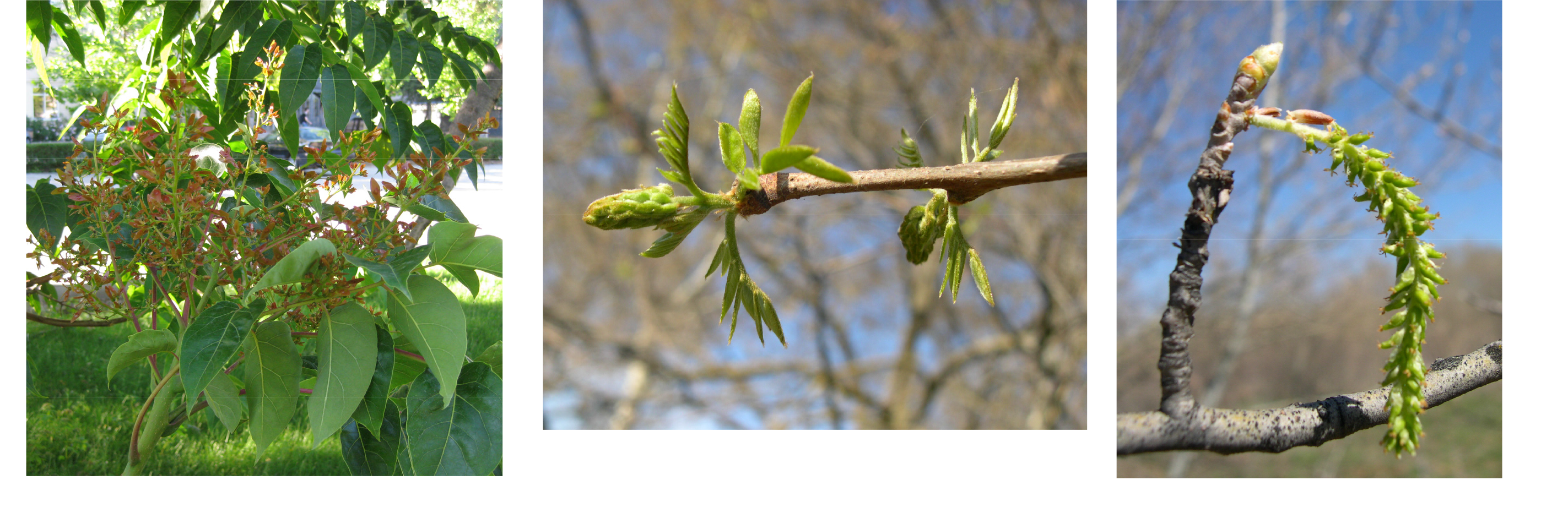

Supplement: Figure S3 — Left- Picture of a current-year shoot of Ailanthus altissima, showing that the same current-year stem bears leaves and inflorescences, which were derived from the same VR bud (see text) (April 2009). Centre- Picture of two recently opened buds of Robinia pseudoacia. It can be observed that both leaves and inflorescence derived from the same VR buds (March 2009). Right- Picture of a female inflorescence of Populus alba. It can be seen that the inflorescence grew on the previous-year stem from a R bud, while the apical vegetative (V) bud, enclosing the current-year vegetative organs, is still close (March 2009). Pictures by the authors. (TIF) [file pone.0100254.s003.tif]
