# Supplementary material for: Can the Life-History Strategy Explain the Success of the Exotic Trees Ailanthus altissima and Robinia pseudoacacia in Iberian Floodplain Forests?
Source: PLoS One. 2014 Jun 17;9(6):e100254. doi: 10.1371/journal.pone.0100254 (PMC4061096; doi:10.1371/journal.pone.0100254)
Supplement: Table S4 — Comparison of shoot production between sexes in Ailanthus altissima. (DOC) [file pone.0100254.s007.doc]

|  | Sex | | DBH | |
| --- | --- | --- | --- | --- |
|  | F-value | *P*-value | F-value | *P*-value |
| *S_t_/S_t-1_* | 0.027 | 0.874 | 1.445 | 0.264 |
| *L_t_/S_t-1_* | 0.980 | 0.351 | 0.049 | 0.831 |
| *FL_t_/S_t-1_* | 3.380 | 0.103 | 0.074 | 0.793 |

**Table S4**. Results of the linear mixed model assessing the effects of sex and stem diameter at breast height (DBH) on the production of on current-year stem (*S_t_*), leaf (*L_t_*), inflorescence (*FL_t_*) and infrutescence (*FR_t_*) biomass per unit of previous-year stem mass (*S_t-1_*) in *Ailanthus altissima*. Tree was included as random factor. Data of the 2011 collection.
